# Burnout and Professional Quality of Life Amongst Crisis Hotline Responders: A Cross-Sectional Survey in Canada During COVID-19

**DOI:** 10.3390/healthcare13091025

**Published:** 2025-04-29

**Authors:** Stephen Lee-Cheong, Mariam Alaverdashvili, Mackenzie Jardine, Vidhi Shivani Maharaj, Nathan Kolla

**Affiliations:** 1 Department of Psychiatry, University of Saskatchewan, Saskatoon, SK S7N 0W8, Canada; 2 Department of Public Health, King’s College London, London SE1 9NH, UK; 3 College of Medicine, University of Saskatchewan, Saskatoon, SK S7N 5E5, Canada; 4 College of Medicine, Saint James School of Medicine, Park Ridge, IL 60068, USA; 5 Centre for Forensic Behavioural Science, Swinburne University of Technology, Melbourne, Alphington, VIC 3078, Australia

**Keywords:** crisis, suicide, hotline, mental wellbeing, burnout, professional quality of life, CBI, ProQOL

## Abstract

**Background/Objectives:** There is a significant gap in accessibility to mental healthcare in Canada. This study aims to examine the population of Canadian crisis hotline responders and investigate the variables that contributed to burnout and professional quality of life during COVID-19. Crisis hotline responders are hypothesized to be affected by burnout and poor professional quality of life, due to the inherent nature of the job and the widespread negative mental health effects of COVID-19, which are expected to continue even after the pandemic. **Methods:** An online, cross-sectional, mixed-methods survey assessed sociodemographic information, shift-related variables, burnout and related factors, and current support methods utilized by crisis hotline responders across Canada. The open-ended questions helped to more personally reflect participants’ experiences. Data were analyzed using chi-square tests, an analysis of variance, and a regression analysis. **Results:** The survey was completed by 136 participants (78.7% female) with an average age of 39.68. Participants reported relatively high levels of burnout/stress on both the Copenhagen Burnout Inventory and professional quality of life survey. Younger age, less work experience, and working overnight shifts emerged as possible predictors of worse mental wellbeing. **Conclusions:** Findings suggest that Canadian crisis hotline responders require greater support to manage workplace burnout/stress. Nevertheless, conducting comprehensive studies during times when there are no public health emergencies are warranted to understand the full scope of burnout in this population. We offer five recommendations to support the mental wellbeing of responders and improve access to this important public health resource.

## 1. Introduction

There is a significant gap in accessibility to mental healthcare in Canada [1,2]. In 2018, 5.3 million Canadians reported that they needed some form of mental health support in the past year; however, 1.2 million stated that their needs were only partially met, and a further 1.1 million stated that their needs were fully unmet with the need for counselling being the least met need. Lack of available resources, limited number of mental healthcare providers, financial barriers, and stigma have been reported as potential reasons for this crisis [2,3]. Certain populations have been found to be more vulnerable to suicide, including males, those living in rural communities, and ethnic minorities, as found by three separate systematic reviews and meta-analyses [4,5,6]. Lack of access to care is hypothesized to drive the rural–urban difference in suicides, which may also result in a reduced chance of a mental health diagnosis being made [4]. Suicide has been found to be the second leading cause of death among Canadians aged 15–34 years old [7].

As a result of these unmet needs, an important accessible resource is crisis hotlines, which often act as a vital first point of contact for individuals experiencing various biopsychosocial crises. Crisis hotlines are designed to be free and often available 24/7 for anyone requiring immediate support and guidance [8,9,10,11]. The Canada Suicide Prevention Service is a national crisis hotline dedicated to suicide prevention [9]. On 30 November 2023, the government of Canada replaced the old 10-digit suicide hotline number with a new 3-digit (9-8-8) number showing that the government recognizes the importance of crisis hotlines as an accessible means for mental health support [12,13]. It is anticipated that there will be an increased awareness of crisis hotlines after the change, which will subsequently lead to an increase in call volume and necessitate measures to ensure the mental wellbeing of crisis hotline responders.

Studies have found several positive and negative factors impacting mental wellbeing amongst professional healthcare workers (doctors, nurses, etc.), but little research has been performed amongst crisis hotline responders [14,15,16]. Positive factors include compassion satisfaction and job satisfaction. Negative factors include burnout, secondary traumatic stress, and compassion fatigue. Burnout is an occupational phenomenon, recognized by the International Classification of Diseases-11, due to unmanaged chronic workplace stress [17]. It has also been defined as a psychological syndrome of emotional exhaustion, depersonalization, and reduced personal accomplishment among people who do “people work” [18]. Maslach measures emotional exhaustion primarily by depletion of physical energy and fatigue, depersonalization is measured by indifference or a distant attitude toward one’s work in general, and personal accomplishment is measured by one’s feelings of competence and successful achievement in one’s work [19]. Stress itself is defined as the physiological or psychological response to internal or external stressors that influences how people feel and behave, while secondary traumatic stress occurs from secondary exposure to extremely stressful events (hearing about someone’s traumas) [20]. Hearing about others’ traumas is a unique part of being a crisis hotline responder, which is a double-edged sword. It can lead to secondary traumatic stress but can also lead to a feeling of satisfaction for the responder when they feel they have helped another person.

Crisis hotline responders report a significant amount of stress and burnout due to continually dealing with a variety of highly emotional and intense calls, including suicidal ideation, sexual assault, and spousal abuse [10,21,22,23,24,25]. Due to fielding anonymous calls, responders often never know the eventual outcome of a caller’s situation, which again can contribute to burnout through resulting frustration and disillusionment [21,25]. The negative impacts of burnout can result in high rates of responder absenteeism and turnover [21,22]. This results in longer wait times for callers, on average less experienced responders answering calls, and an overall decrease in the quality of support provided to callers [24]. The majority of crisis hotline responders are volunteers rather than trained mental health professionals. The lack of extensive training and experience combined with the stressors of balancing priorities related to work or school puts volunteer responders at an increased risk for burnout [10,21,22,23,24]. As in other professions, more experienced workers act as role models for the inexperienced ones, which can be either positive or negative. Burnt-out responders may end up influencing others to become burnt-out themselves, as they exhibit disillusionment and emotional exhaustion [26].

Gender is a potential risk factor for poor mental wellbeing amongst healthcare workers. It seems that amongst nurses and the general population, that the evidence is mixed, regarding if males or females are at higher risk, or if the risk is equal [8,15,27]. However, for certain healthcare workers, including physicians, psychologists, and psychotherapists, females seem to be more consistently found to be at higher risk of poor mental wellbeing, including during the pandemic [7,28,29,30]. This may be related to more traditional gender roles where females spend more time compared to males as caregivers to children and performing household tasks, which likely became more time-consuming and stressful during a period of lockdown [31]. The way people act, speak, and conduct themselves is also influenced by gender roles [31]. Depending on the mindsets of gender roles in a partnership, this could negatively affect females’ work–family conflicts and play a role in these findings [31].

Previous literature has found variables of increasing age, work experience, and education to predict lower levels of burnout and related stress, including within nurses and physicians [28,29,30]. It has been suggested that this may be related to the fact that younger people are less experienced in handling extreme events; however, there seem to be other factors at play as well [29,30]. In their professional lives, younger workers are more likely to deal with more psychologically and physically demanding jobs, lack of authority at work, job insecurity, and irregular work schedules [27]. In their personal lives, they may be dealing with challenging life events, such as living on their own for the first time, getting their first jobs, being in their first serious romantic relationships, and having young children [27].

New challenges were brought to crisis hotline responders during the COVID-19 pandemic, such as an increase in the volume of calls and a change in the type of calls received, which likely contributed to a heightened burnout level in crisis hotline responders [32]. Pre-COVID-19, calls to crisis hotlines were predominantly reported to be regarding relationship issues (37%), loneliness (20%), or various fears and anxieties (13%) [32]. However, during the first wave of the pandemic, there was a reported increase in calls regarding fear of infection from COVID-19 and loneliness, to which there was a lot of uncertainty, and those concerns were likely shared by responders themselves [32]. Suicide rates are highly sensitive to macroeconomic changes, particularly unemployment, which did increase during COVID-19 [33]. In Canada, suicidal ideation was increased in those experiencing unemployment or worsening socioeconomic status, and monetary inflation post-COVID-19 has continued to cause economic difficulties that may perpetuate this problem [33]. The pandemic also forced many responders to work from home, thereby reducing their access to support and increasing their risk of burnout [14]. Worldwide, healthcare workers dealt with increasing patient demands, complex health conditions, workforce shortages, administrative burdens, and long hours, which all lead to emotional stress [26]. In keeping with the described changes during COVID-19, certain shift-related factors, such as average hours worked per month, average length of day shift (hours), average length of overnight shift (hours), and average number of overnight shifts per month, were hypothesized to be associated with differences in the mental wellbeing of responders.

Previous national studies on burnout in crisis hotline responders have produced mixed results, as a 2017 study from the UK found that, compared to population norms, UK responders had lower levels of emotional exhaustion and depersonalization and moderate to high personal accomplishment, while a 2016 Australian study found that 1 in 4 participants reported moderate to very high symptoms of general psychological distress [34,35]. To date, no study has investigated the national proportions of burnout amongst crisis hotline responders in Canada. The aim of this study was to conduct a cross-sectional survey regarding burnout and professional quality of life amongst crisis hotline responders across Canada, during the COVID-19 pandemic. This included measuring burnout, secondary traumatic stress, compassion fatigue, and compassion satisfaction; and analyzing which variables were significantly associated with each. We hypothesized that proportions of burnout would be high and professional quality of life would be low considering the widespread national challenges faced by Canadians during the pandemic. We hypothesized that gender, age, work location, work experience, shift start time, and number of overnight shifts would be associated with differences in CBI and ProQOL subscale scores.

## 2. Materials and Methods

### 2.1. Study Design

A cross-sectional assessment by questionnaire consisting of sociodemographic information, shift-related variables, measures on burnout and related factors, organization of crisis hotlines, and responder training was administered online through SurveyMonkey from November 2021 to May 2022. A mixed-methods design was implemented, which allowed for comparison of measure scores and the opportunity to capture unique experiences/responses in the open-ended questions. The methodology of this study was informed by the STROBE guidelines for cross-sectional studies [36].

Inclusion criteria were the following: current responder at a crisis hotline in Canada, ≥18 years old, proficient in English or French, and has been taking unsupervised calls for >30 days.

### 2.2. Sample

A minimum sample size (n = 73) was determined for linear regressions, with a medium effect size 0.15, alpha error probability 0.05, power 0.90, and 1 predictor. Calculations were performed using G*Power software Version 3.1.9.6. The test family was specified as F tests, with the analysis type set to linear multiple regression: fixed model, R^2^ deviation from zero. The total sample size for the quantitative component was 136.

We did not determine a qualitative sample size at the beginning of the study, as it relies on reaching saturation. The total sample size for the qualitative component was 121.

To reduce bias and obtain the largest sample possible, the authors invited all crisis hotline centres from every province and territory of Canada. Both English and French versions of the questionnaire were disseminated. Originally, only volunteers were invited but due to a low response rate, the invitation was also extended to paid staff.

Every crisis hotline that agreed to participate (n = 58) indicated that they were short-staffed. Three crisis hotlines stated they would not participate due to being short staffed, the responders’ increasing workload, and a desire for the responders to focus on responding to calls given the COVID-19 pandemic. Two crisis hotline managers indicated that they did not want to participate as they themselves were feeling too burnt-out. Nine crisis hotlines did not respond at all. Overall, 58/72 centres invited agreed to participate, but only 22 centres (10 urban) had responders who participated. None of the territories participated, and 109/136 participants worked for crisis hotlines in major urban centres.

### 2.3. Sample Characteristics

In total, 22 of 72 invited crisis hotline centres participated, which yielded 136 responders who completed the cross-sectional assessment. A total of 8 out of 10 provinces participated, and none of the territories participated. Sixteen responses were in French. The majority of participants were female (78.7%), and the ethnicity participants identified as the most was Caucasian (75.89%) followed by East Asian (5.67%) and South Asian (5.67%). Ontario had the most participants (40%), followed by Alberta (20.7%). The average age of participants was 39.68, with ages ranging from 20 to 76 years old. The majority of participants worked on their crisis hotline in English (86.7%), 11.1% worked in French, and 2.2% alternated between English and French. A more complete breakdown of these data is found in Table 1. The majority of participants had an education level above high school (97.8%), and 31.6% had completed some or all of a graduate degree. Participants mostly had a healthcare and social sciences education (55%), and while a variety of current professions were reported, many were again from healthcare and social sciences (including professional crisis hotline responders) (24%). A significant proportion of participants reported that they were students (28%) or retired (13%) (41% total), which likely accounts for the discrepancy between those with an education in healthcare and social sciences versus those currently working in those fields. A minority of participants (8%) were crisis hotline employees rather than volunteers. During the COVID-19 pandemic, 66 crisis hotline responders worked from home, 51 worked from an office, and 19 alternated. Prior to the pandemic 2 crisis hotline responders worked from home, 55 worked from an office, and 1 alternated, while 78 were not employed as a crisis hotline responder.

### 2.4. Measures

Sociodemographic data were collected based on previously reported associations for burnout in crisis hotline responders, including age, educational background, and occupation (volunteer or professional crisis hotline responder) [22,23].

Two measures of burnout and related factors were administered to participants in an attempt to provide a more comprehensive understanding of the experiences they had, including both positive and negative aspects.

The Copenhagen Burnout Inventory (CBI) is a 19-item survey [32]. A total of 13 items (6 personal and 7 work related) relate to physical and psychological fatigue/exhaustion, and 6 client-related items relate to burnout. The three subscales, “Personal Burnout”, “Work-related Burnout”, and “Client-related Burnout”, are scored from 0 to 100. Scores > 50 are considered a ‘high degree of burnout’ [37]. Examples of items are “How often do you feel worn out?” (Personal Burnout), “Are you exhausted in the morning at the thought of another day at work?” (Work-related Burnout), and “Are you tired of working with clients?” (Client-related Burnout). CBI scales have high internal reliability and are validated for use in any adult occupational group in any setting [37,38,39,40,41]. The validity and reliability of the CBI was analyzed on the basis of a prospective study of burnout in employees in the human service sector, the 2005 PUMA study (Project on Burnout, Motivation, and Job Satisfaction) [37,38]. The Copenhagen Burnout Inventory (English Version) was used for English responders, and the French version “Inventaire de burnout de Copenhague” was used for French responders.

The ProQOL is a 30-item survey that measures a participant’s compassion satisfaction and compassion fatigue, as a result of their work situation and life in general in the last 30 days [42]. The three, 10-item subscales, “Compassion Satisfaction Scale”, “Burnout Scale”, and “Secondary Traumatic Stress Scale”, are initially scored from 10 to 50, then are converted to t-scores for analysis, and further categorized as “low” (<43), “average” (around 50), and “high” (>57) [42]. Compassion fatigue informs impairment in job satisfaction, emotional and physical health, productivity, quality of work, customer/patient satisfaction, and increased levels of staff turnover, and is measured by the burnout and secondary traumatic stress scales. In contrast, compassion satisfaction is a measure of the pleasure one derives from doing their work well and is a factor that can help prevent burnout and secondary traumatic stress. Examples of items are “I am happy that I chose to do this work.” (Compassion Satisfaction), “I feel “bogged down” by the system.” (Burnout), and “I can’t recall important parts of my work with trauma victims.” (Secondary Traumatic Stress). The ProQOL has a history of high reliability scores and good construct validity [42,43]. ProQOL Version 5 in English and French were used.

Thirteen custom-made questions were designed based on the existing literature to determine how the organization of crisis hotlines and specifically their training methods were supporting responders (e.g., Does the rigidity of your crisis hotline schedule regularly cause you stress? Do you have access to ongoing training on how to manage calls? Are you able to debrief with workplace peers after a difficult call?) (Table S1) [10,11,21,22,23]. An additional custom-made question asked participants to list their top five choices in no particular order of support methods viewed as being most helpful to mitigate burnout/stress (Table S1). To avoid stereotyped response patterns, the question order for the Copenhagen Burnout Inventory (CBI) was mixed, considering the CBI instructions [37].

Awareness, utilization, and desire of various support and training methods for crisis hotline responders was assessed through custom-made questions and is summarized in Table S1.

Data were collected on average shift start time, as it might be a possible contributing variable to burnout and related factors. We considered time of day to be categorical data as a higher number on a 24 h clock does not necessarily infer a ‘better’ time. The shift times were therefore grouped by 4 h inclusive windows (8–11 am, noon–3 pm, 4–7 pm, 8–11 pm, midnight–3 am, 4–7 am) in order to perform the analyses.

To further understand participants’ experiences with mental wellbeing, they were asked open-ended questions, but no interviews were conducted. Question #1: “Which of the support methods available to you do you feel help the most and why?” Question #2: “What do you think contributes the most to your stress as a crisis hotline responder?” Question #3: “How do you feel you could be better supported as a crisis hotline responder? If you feel that your support level is good, please state why.” Question #4: “How has burnout affected your ability to be a crisis hotline responder?” Question #5: “Do you have any other comments you would like to add which have not been discussed?”

The questionnaire survey was piloted for feedback with one volunteer crisis hotline responder in a think-aloud interview with appropriate suggestions implemented, such as inclusion of different employment categories, rewording of questions, and exclusion of certain questions.

### 2.5. Data Collection

Crisis hotline centre managers were contacted through email/telephone. The managers informed their responders of this study opportunity and provided an information sheet that included a link to sign up if interested. A questionnaire was then sent that included the information sheet and a consent form. Informed consent was obtained from all participants involved in the study, including permission to publish their anonymized data. A reminder email was sent out 14 days later for non-responders and partial responders. Withdrawal was possible at any time during the questionnaire and up to seven days after it closed. Four participants withdrew during survey administration and zero withdrew after they had finished responding.

### 2.6. Data Analysis

In cases where a participant had partially or completely missing data for a subscale of the CBI and/or ProQOL, those participants were omitted from the analysis for that particular subscale. The internal consistency reliability of the CBI and ProQOL subscales were determined by calculating Cronbach’s α. Two-way t-tests were performed regarding the associated differences for gender on CBI and ProQOL subscale scores. One-way ANOVA analyses were performed for work environment, average shift start time, and age groups against the CBI and ProQOL subscale scores. A Tukey–Kramer test for post hoc analysis was performed when the initial result was statistically significant as group sizes were different. Linear regressions were performed for variables (age, work experience in months, average hours worked per month, average length of day shift in hours, average length of overnight shift in hours) against the CBI and ProQOL subscale scores.

Regarding the open-ended questions, thematic analysis was performed in accordance with Braun & Clarke (2006) by using their 6-step, non-linear, recursive process [44]. The analysis was inductive and semantic, allowing themes to emerge from participants’ open-ended responses without applying a pre-existing coding framework. The analysis was carried out manually by the primary researcher, who engaged in reflexive practices to ensure thoughtful interpretation of the data. This was performed in an attempt to explore the participants’ experiences and help develop explanations as to why they occurred [45]. SPSS Version 28.0.1.0 was used to perform the quantitative analyses. NVivo 12 was used to perform the thematic analysis of the open-ended questions.

## 3. Results

### 3.1. What Were the Burnout and Professional Quality of Life Measure Scores?

CBI personal, work-related, and client-related burnout mean scores were found to be below what is considered a ‘high degree of burnout’ (>50 points) (Table 2).

Compassion satisfaction and burnout mean scores for the ProQOL were within the ‘average’ level of scoring, while the secondary traumatic stress mean score was within the ‘high’ level of scoring (Table 2). Compassion satisfaction mean scores were on the high end of average, and the majority of participants scored in the average or high levels (41.2% and 48.9%, respectively). On the burnout scale, most participants scored in the average level (65.6%), and an almost equal amount scored in the low and high levels (16.8% and 17.6%, respectively). Secondary traumatic stress scores were high (38.2% average and 61.8% high), with no participants scoring in the low level.

### 3.2. How Did CBI and ProQOL Subscale Scores Relate to Each Other?

Pearson’s correlation coefficients were calculated between each pair of subscales. ProQOL compassion satisfaction showed a moderate negative correlation with CBI personal burnout (r = −0.421, *p* ≤ 0.001) and work-related burnout (r = −0.689, *p* ≤ 0.001), and a weak positive correlation with client-related burnout (r = 0.094, *p* = 0.299). ProQOL burnout showed a moderate positive correlation with CBI personal burnout (r = 0.510, *p* ≤ 0.001) and work-related burnout (r = 0.648, *p* ≤ 0.001), and a weak negative correlation with client-related burnout (r = −0.036, *p* = 0.691). ProQOL secondary traumatic stress showed a moderate positive correlation with CBI personal burnout (r = 0.535, *p* ≤ 0.001) and work-related burnout (r = 0.519, *p* ≤ 0.001), and a weak positive correlation with client-related burnout (r = 0.159, *p* = 0.078).

### 3.3. How Did Different Variables Correlate with CBI and ProQOL Subscale Scores?

Two-way *t*-tests were performed regarding the associated differences in gender on CBI and ProQOl subscale scores. Three participants identified as non-binary and one identified as gender neutral, which we excluded from this analysis considering the extremely small sample size. On the CBI Personal Burnout Scale, females (M = 45.95, SD = 20.21) scored statistically significantly higher than males (M = 35.5, SD = 13.51), t(129) = 2.46, *p* = 0.015. On the CBI Work-Related Burnout Scale, females (M = 42.92, SD = 17.88) scored statistically significantly higher than males (M = 34.71, SD = 17.21), t(129) = 2.08, *p* = 0.039. On the ProQOL Secondary Traumatic Stress Scale, females (M = 61.02, SD = 9.13) scored statistically significantly higher than males (M = 56.67, SD = 6.21), t(124) = 2.21, *p* = 0.029. The rest of the subscales were not found to have any statistically significant gender-based differences.

Age was the only demographic variable found to have a statistically significant difference on all of the CBI and ProQOL subscale scores (Table 3). Younger individuals were found to suffer more from burnout and secondary traumatic stress and had lower compassion satisfaction (Table 3).

Work experience was found to have a statistically significant difference in the CBI Client-Related Burnout subscale and all of the ProQOL subscales (Table 3). Those with more experience were found to have lower levels of burnout on the CBI Client-Related scale and ProQOL, lower levels of secondary traumatic stress, and higher compassion satisfaction.

The greater number of overnight shifts completed per month was found to have a statistically significant difference on increasing scores for the CBI Personal-Related and Client-Related Burnout subscales and the ProQOL Secondary Traumatic Stress Scale (Table 3).

A follow-up Tukey–Kramer test was performed for the results from Table 4, which yielded statistically significant differences on the different measured subscales as described below.

**Table 4:** The associated differences in age groups on CBI and ProQOL subscale scores by one-way ANOVA. Ages were divided into four categories based on Statistics Canada’s framework related to the labour market [46]. The categories were 18–24, 25–54, 55–64, and 65+.

Subscales	ANOVA Result
CBI Personal Burnout	F (3, 119) = [6.26], *p* = 0.001
CBI Work-Related Burnout	F (3, 119) = [8.50], *p* = 0.001
CBI Client-Related Burnout	F (3, 115) = [10.00], *p* = 0.001
ProQOL Compassion Satisfaction Scale	F (3, 119) = [4.38], *p* = 0.006
ProQOL Burnout Scale	F (3, 123) = [5.55], *p* = 0.001
ProQOL Secondary Traumatic Stress Scale	F (3, 119) = [0.97], *p* = 0.410

CBI Personal Burnout Scale: the 18–24 age group scored significantly higher than the 55–64 age group (mean difference 17.37, 95% CI: 3.88–30.86, *p* < 0.001) and the 65+ age group (mean difference 20.36, 95% CI: 5.57–35.15, *p* < 0.001), and the 25–54 age group scored significantly higher than the 65+ age group (mean difference 13.71, 95% CI: 0.06–27.36, *p* < 0.001).

CBI Work-Related Burnout Scale: the 18–24 age group scored significantly higher than the 55–64 age group (mean difference of 20.82, 95% CI: 8.68–32.96, *p* < 0.001) and the 65+ age group (mean difference 15.88, 95% CI: 2.57–29.19, *p* < 0.001), and the 25–54 age group scored significantly higher than the 55–64 age group (mean difference 15.92, 95% CI: 4.91–26.92, *p* < 0.001).

CBI Client-Related Burnout Scale: the 18–24 age group scored significantly higher than the 55–64 age group (mean difference 21.80, 95% CI: 10.31–33.29, *p* < 0.001) and the 65+ age group (mean difference 18.41, 95% CI: 5.83–30.99, *p* < 0.001), and the 25–54 age group scored significantly higher than the 55–64 age group (mean difference 13.47, 95% CI: 3.05–23.88, *p* < 0.001).

ProQOL Compassion Satisfaction Scale: the 18–24 age group scored significantly higher than the 55–64 age group (mean difference 7.17, 95% CI: 1.26–13.09, *p* < 0.001).

ProQOL Burnout Scale: the 18–24 age group scored significantly higher than the 55–64 age group (mean difference 7.34, 95% CI: 1.56–13.12, *p* < 0.001) and the 65+ age group (mean difference 7.79, 95% CI: 1.81–13.77, *p* < 0.001).

One-way ANOVAs were performed, which did not yield statistically significant differences between different work locations (office, home, alternating between home and office) on CBI and ProQOL subscale scores, but did for differences in work shift start time on the ProQOL Secondary Traumatic Stress Scale (F(5, 124) = [2.53], *p* = 0.032). However, a follow-up Tukey–Kramer test did not yield a statistically significant difference.

### 3.4. How Did Participants Engage with Professional Support and Training Modalities?

The majority of study participants acknowledged available assistance and supportive resources, especially training opportunities on setting limits/boundaries with callers and possibilities to debrief with supervisors after a difficult call (Table S1). Although clear guidelines and procedures to ensure consistency and efficiency in handling calls was recognized by study participants as important, flexible policies regarding shift scheduling was the most often selected strategy viewed to help mitigate burnout/stress (n = 83). When asked “Does the rigidity of your crisis hotline schedule regularly cause you stress?”, 65.7% responded “Yes”, 29.1% responded “No”, and 5.2% responded “I don’t know” (Table S1). Training, promoting, and supporting self-care (n = 62) was the second most selected of these strategies (n = 63). When asked “*Did your training involve a component on recognizing and managing burnout?*”, 58.1% responded “Yes”, 30.1% responded “No”, and 11.8% responded “I don’t know” (Table S1).

### 3.5. What Were Participants’ Insights on Burnout/Stress and Related Supports?

The open-ended questions were included to more personally reflect participants’ unique nuanced experiences. A thematic analysis was performed for each question, as described in Table S2. Another thematic analysis was performed based on the themes identified from those questions to more holistically describe participants’ experiences as crisis hotline responders in relation to their mental wellbeing. Four main themes emerged, which are described below and are supported by direct quotes from some of the responses.

Theme 1: Support from colleagues

***Support from staff***

This was the most commonly reported available support method that participants felt was helpful to them and was also the one they reported as being the most desirable. Some participants indicated they felt staff were kind and approachable, helping to alleviate some of the burden of responsibility that participants, specifically volunteers, felt. In the same vein, they also voiced a desire to have improved relationships with staff, as others felt they received harsh criticism and felt unsupported when voicing concerns over difficult callers. The ability to debrief with staff after a difficult call, receive real-time feedback, and have their own feelings/emotions managed was highly desired. Some stated they had access to a mentor for ongoing learning and support, sometimes by shadowing live calls, while others did not.

“*I feel the most supported when getting feedback from my supervisors, and positive reassurance/validation that I am a good call taker.*”

(Participant 1, P1)

“*Volunteers also need different levels of support when they are new versus when they have been doing the work for a while. It would make a huge difference if I could at least observe seasoned volunteers, esp. supervisors, handle calls. It would also be super helpful if I could have a performance review once in a while (maybe after x number of volunteer hours) to receive feedback on how I’m doing.*”

(P25)

***Peer support***

This was the second most commonly cited available support method that participants felt was helpful to them. Participants seemed to enjoy the comradery built amongst their peers and at times felt safer to discuss challenges without fear of judgement from their peers. However, the impact of COVID-19 made it difficult for others to remain connected to their peers, who felt they had lost their sense of community and ability to receive support. The high turnover of responders at baseline and the overall decline in active responders during COVID-19 left some participants feeling overwhelmed by the amount of calls they were required to answer.

“*Hearing the similar experiences of peers during check-ins is helpful because it feels like I am not the only one who experiences feelings of anxiety.*”

(P38)

“*We used to have sharing nights where we would talk about our difficulties as volunteers—I really miss that sense of community. Losing our sense of community has made volunteering infinitely more difficult. The only support method we currently have is calling a peer. It helps, but is not enough.*”

(P23)

Theme 2: Feelings of inadequacy

***Further training***

The majority of participants were volunteers, which played a significant role in this theme. Participants consistently voiced not feeling self confident in their role as a crisis hotline responder. The fear of not doing a good job and the weight of the responsibility they felt seemed to often stem from a lack of ongoing training. There were, however, many participants that stated they felt their level of training was adequate and that they had access to ongoing training.

“*Not knowing if I could do more/if I’m doing well—taking crisis calls is a HUGE responsibility, and often I feel like volunteers have the same level of responsibility as full-time professionals, and are expected to know social services as much as professionals. We’re not! How can I?*”

(P86)

“*Support level is so good. Consistent check ins on shift and then also monthly meetings with my training shift supervisor, plus everyone in the organization is willing to help support you.*”

(P42)

*Unpredictability*

Many participants stated that not knowing what was going to happen on their next call was extremely difficult for them to manage. This seemed to be connected to feelings of inadequacy as the participants wondered if their abilities would be enough to help the caller.

“*The unpredictable nature of calls and not knowing what is waiting for you when you pick up the phone and if you have the skills to help that caller.*”

(P2)

“*When someone ends the line and you don’t know if they will be safe.*”

(P44)

***Difficult callers***

Participants voiced feelings of inadequacy when it came to dealing with difficult callers. The most common types of difficult callers were repeat callers, who often spoke about the same things, abusive callers, and callers with complex issues.

“*I talk to the same 3 or 4 people on the lines about the same handful of issues. Yet the training modules do not always apply…I sometimes feel pessimistic about social services in general and this heart break is exhausting to endure.*”

(P97)

“*The very high number of calls we receive that involve abuse towards the volunteer or sexual harassment towards the volunteer. It is an issue we have raised to the administration many times and I feel we have no support in this matter. The attitude seems to be that we need to just “deal with it”.*”

(P23)

Theme 3: Schedules

***Rigidity***

Some participants stated they felt the scheduling at their crisis hotline was fair and helpful for them, while others stated that rigid scheduling was having a direct negative impact on their lives. The demands to attend promptly to high call volumes was challenging for those with their own mental health struggles, leaving some feeling unappreciated for their efforts. Some stated that they felt attitudes from staff were negatively impacting them.

“*Flexible schedule—At my centre, after the first year of volunteering, we are given more flexibility with our shifts…This helped me plan my life, and give balance. Weekly shifts were too draining.*”

(P106)

“*I honestly find the 4 h shifts a little too long. At the 2–2.5 h mark it can become exhausting because of how much you give to callers—especially now during the pandemic where call volume has increased and perhaps volunteer call-takers have not.*”

(P45)

***Overtime***

The requirements of overtime shifts were consistently viewed in a negative light, particularly as volunteers had to juggle the demands of their regular jobs. This type of shift was unique in that some participants voiced preferring to do it from home.

“*I also think that mandatory 50 h of overnight shifts in person is a really big ask for volunteers…I felt a lot less burnt out from volunteering after I was able to finish my overnight shifts.*”

(P12)

What was thought as contributing the most to stress as a crisis hotline responder was *“…managing the rigidity of how the crisis line has structured the volunteer commitments and my own personal life and other commitments; driving before or after an overnight shift…”* (P12)

Theme 4: Impact of working on participants

***Unable to provide best care***

Many participants voiced the inability to be as empathetic with patients as they used to be and that they were not able to provide their best efforts in supporting the caller. Some also stated that this had caused them to not attend their shifts as scheduled and/or consider quitting. Fatigue and increasing irritability were the most common ill effects experienced in relation to anticipating, actively, or having just finished working.

“*It has made me feel more jaded and irritated when talking to callers. I should be more caring and empathetic, but when you are constantly irritated that is very difficult. Some days I dread picking up the phone, knowing that it is a 50/50 chance I’m going to get berated over the phone by the caller.*”

(P1)

“*I feel that burnout prevented me from being able to be the call taker that I wanted to be and that the callers deserved. It has also caused me to call in sick for shifts or make up excuses not to go simply because I was too exhausted (from both volunteering and many other commitments).*”

(P12)

***Physical and mental health***

Many participants stated that they did not struggle with burnout or other ill effects from being a crisis hotline responder, which was attributed to sufficient supports and flexible scheduling. However, some had negative impacts on their physical and mental health, which spilled over into their personal life.

“*No (I don’t suffer from burnout). I could see how this job long term might cause burnout if supports are not in place.*”

(P42)

“*I feel as though I am stressed, on edge, short tempered and when I go home, I feel so fatigued that I have no energy to enjoy my usual hobbies and often stay at home because I do not have the energy to go out and socialize as I normally would.*”

(P126)

### 3.6. Thematic Differences Across Age and Gender

Chi-square independent tests were calculated to determine if there were statistically significant differences regarding age categories or gender and the different themes identified for open-ended questions #1–4.

Analyses for questions #1–3 did not indicate any statistically significant differences among age groups or gender. For question #4, “how has burnout affected your ability to be a crisis hotline responder?”, the analysis indicated a statistically significant difference among age groups, X^2^ (6, N = 99) = 15.51, *p* = 0.017, but not for gender. The breakdown of responses to this question by age group is reported in Table S3 for further explanation.

Post hoc analysis of standardized residuals revealed that the 18–24 age group was statistically significantly more likely to report being unable to provide the best care due to burnout and less likely to report no burnout. The 55–64 age group was statistically significantly more likely to report no burnout and less likely to indicate that burnout affected their ability to provide care. The 25–54 and 65+ age groups did not show statistically significant deviations from expected response patterns.

## 4. Discussion

Crisis hotline managers, including the ones that agreed to have their responders participate, consistently indicated that they were short-staffed during the data collection period as responders had quit or gone on a leave of absence due to the increasing levels of stress both at the crisis hotline and in their personal lives. This was unsurprising, as other healthcare providers also experienced a high incidence of burnout during the pandemic [28,29,30]. The degree of secondary traumatic stress, which is work-related secondary exposure to extremely stressful events, also approached a high threshold in our study participants [47]. The previously mentioned profession-related challenges due to more complex and demanding phone calls during the pandemic might be one of the reasons for this. There was a five-times-higher PTSD rate, related to COVID-19, seen in physicians during the pandemic, which was explained by the sharp increase in exposure to more severe cases of COVID-19 and related traumatizing experiences [28]. A high percentage of participants from our study suffered from high degrees of secondary traumatic stress and a low percentage had a low level of burnout. All of this highlights the intensive use of crisis hotlines during the COVID-19 pandemic and implies the need for the implementation of additional or continuous training beyond the standard practices and/or more frequent check-ups/debriefs in times of public health emergencies for crisis hotline responders.

Study participants had high levels of compassion satisfaction (the pleasure one derives from doing their work well) [16]. They reported their satisfaction and fulfilment were derived from being able to help and make positive changes in others’ lives during difficult times, despite the burnout and secondary traumatic stress they suffered from themselves, which is a finding consistent with previous literature [25]. In addition to the negative side effects of secondary traumatic stress, it should be noted that positive changes can also occur, such as vicarious posttraumatic growth [15]. This phenomenon has been described, particularly in healthcare workers, which helps individuals adapt to the transformations in their life and reconsider the way they see the world in domains, such as personal strength, new possibilities, relating to others, spiritual and existential change, and appreciation of life [15]. Those that responded to the study during such a stressful period may have represented a uniquely resilient cohort, which would increase the levels of compassion satisfaction captured and reduce the levels of burnout captured. In kind, severely affected participants may have already quit/gone on a leave of absence or not responded to the survey due to burnout, which would have underestimated our results in terms of burnout and secondary traumatic stress.

The Pearson’s correlation coefficient calculations revealed that the two measures’ subscales had, in predictable directions, moderate effects on one another, except for CBI Client-Related Burnout. This may speak to the ability for crisis hotline responders, like other healthcare workers, to compartmentalize their mental health struggles and put their callers’ needs ahead of their own, which may actually worsen their mental wellbeing [48]. However, despite the anonymity of the survey, social desirability biases may still have occurred in this self-reported surveys and lead to an underestimation of burnout, particularly in the CBI Client-Related Burnout Scale [26].

Females were found to score statistically significantly higher on three subscales, the CBI Personal and Work-Related Scales, and the ProQOL Secondary Traumatic Stress Scale. In contrast to nurses, female physicians, psychologists, and psychotherapists seem to more consistently be at higher risk for negative mental health outcomes [15,27,28,29,30,49,50]. This may be related to the increased levels of responsibility that they bear. Similarly, our participants often voiced feeling immense pressure as they often had no one to turn to during a call. The participants for the two previous national studies were mostly females, with 69.1% for the UK and 78.1% for Australia, which is relatively consistent with the proportion of females working as mental health professionals in those respective countries [34,35]. Canada is similar in this trend of having predominantly females working in mental health [51]. In 2021, it was found that in Canada, 77% of psychologists, 82% of psychotherapists/counselling therapists, and 82% of registered psychiatric nurses were female. Although the high proportion of female participants may limit the generalizability of our findings, considering the similar overall proportion of female mental health workers in Canada, this reinforces the validity of our sample.

Younger age and less work experience emerged as possible risk factors contributing to increased levels of client-related burnout, compassion fatigue, and lower levels of compassion satisfaction. Younger age emerged as a possible risk factor contributing to increased levels of personal and work-related burnout (Table 4). The age group of 18–24 was consistently found to have worse outcomes than the other age categories. Previous literature has suggested that younger people are less experienced in handling extreme events and so are at increased risk of burnout and stress [29,30]. It has been found that lack of perceived competence is actually the true factor in relation to these variables, according to studies regarding psychological factors causing burnout or work-related stress [16,48,50]. Nonetheless, younger age is still likely a good indicator of lower perceived competence based on the factors we listed above. Many of our participants were students, which may increase the significance of the associated difference in age on burnout in this population as the youngest crisis hotline responder will be younger than the youngest employee in other populations such as nurses or physicians.

Shift start time was hypothesized to have potentially had an associated difference on mental wellbeing, due to various factors such as: the volume, type, and intensity of calls; the levels of support available; and the fatigue levels of the responders. However, no statistically significant difference was found between shift start time and CBI or ProQOL scores. The number of overnight shifts performed per month was found to have a statistically significant difference on increasing the levels of personal and client-related burnout, as well as secondary traumatic stress. It is possible that the reduced level of support at night time, coupled with the increasing physiological demands of working at late hours, while still having a day job is what explains these findings [52]. The open-ended questions revealed that the length of overnight shifts was likely a significant component of this finding, despite the open-ended questions not being able to find any statistically significant differences amongst those who did overnight shifts, likely due to the small sample size (n = 43) and the relatively similar length of overnight shifts.

Despite the overall heightened proportions of stress and burnout amongst crisis hotline responders in our study, many participants did not score high on either the CBI or ProQOL. CBI results from the current study were compared to the data derived from a 2022 Swedish study on psychotherapists, considering the similarities in work in another Western country [50]. We would have preferred to compare our CBI scores to a sample representative of the general population of Canada or of a population of similar professions in Canada. However, we were unable to find such data. On all CBI subscales, the current study participants were found to have scored below what is considered a ‘high degree of burnout’ (>50 points) but still scored higher than the Swedish study (Table 2). Several factors may help reduce burnout amongst professional psychotherapists, including older age, more advanced training, more years of experience, and perhaps most importantly, perceived competence [50].

ProQOL results were compared to average scores provided by the ProQOL manual [42]. The current study participants were found to have higher rates of compassion satisfaction and secondary traumatic stress and nearly equal burnout rates. From the ProQOL manual, the scores were the same for each subscale, which reported a mean of 50 with standard deviation of 10 (n = 1187). For compassion satisfaction, the current study participants scored 55.14 with standard deviations of 7.97 (n = 132). For secondary traumatic stress, the current study participants scored a mean of 59.92 with standard deviation of 7.00 (n = 131). For burnout, the current study participants scored a mean of 49.59 with standard deviation of 7.80 (n = 131).

All of the main themes identified in the open-ended questions seem to be interwoven, with each influencing and being influenced by one another. The desire for increasing amounts of staff and peer support were nuanced by the fact that participants often voiced a lack of self-confidence in their abilities, and an accompanied desire for further training to help them manage difficult and unpredictable situations, such as violence/sexual abuse, aggression, manipulation, and complex mental health/social problems.

Although our results did not yield any statistically significant difference in CBI or ProQOL scores related to working at home or in an office, in future times, where working from home and isolation is required, the increased risk for poor mental wellbeing should be considered and monitored. In the open-ended questions, our participants voiced concerns over isolation, while working from home, which reduced their ability to connect and support one another. Responders seemed to feel trapped by their overall situation, which was amplified by the rigidity of schedules that had negative impacts on their personal and professional lives. These negative impacts affected participants’ abilities to provide the best possible care for callers while they were working and even affected their ability to attend to their crisis hotline and continue on in their role as a responder.

The open-ended questions indicated a significant proportion of participants cited that they felt their level of support received was adequate and that they did not suffer from burnout. This may be attributed to good organizational infrastructure, including effective training and supportive work environments. Awareness of available resources and participation in training opportunities seems to strengthen crisis hotline responders’ resilience. Nevertheless, some discrepancies between researcher-posed questions (i.e., quantitative questions) vs. responder-initiated themes (i.e., open-ended questions) indicate the need to further refine the organizational infrastructure of crisis hotlines, especially during a public health crisis. Flexible shift arrangements for crisis hotline responders seems to be a crucial aspect to protect them from excessive job-related burnout and may help to retain them for longer and prevent future staffing shortages [25,53]. Furthermore, in addition to improving the training and support provided, increased promotion of supports that are already available seems necessary. Many participants reported they were unaware of their availability yet voiced their desire for same.

Crisis hotlines can help to mitigate burnout by creating supportive work environments that foster resilience in their employees, through things such as the promotion of teamwork and the optimization of work schedules [26]. Responders have their own role to play as well in mitigating burnout, as they would benefit from being proactive in prioritizing their physical health, social health (including connecting with colleagues), and engaging in self-reflection with continuing education [26]. As initially suggested by the literature and corroborated by our data, the implementation of a ‘buddy system’ that pairs experienced responders with less experienced ones seems beneficial, as it was one of the support methods most often selected by participants as being desired while simultaneously being one of the least available [21,54]. This would allow for shadowing of live calls, ongoing training, and an avenue for peer support and peer bonding. As the challenges faced by responders have proven to be dynamic based on global factors, this ‘buddy system’ may provide an avenue for more up to date feedback on what responders require for support.

### 4.1. Limitations

The limitations of a cross-sectional survey apply, including risk of participant recall bias, and the fact that the data can only imply association but not causation. The use of a self-reported survey introduces the potential for recall and social desirability biases and may even lead to an underestimation of burnout [26]. The majority of the study took place during the height of the COVID-19 pandemic, which may have affected the sample size, which was not as large as in other pre-pandemic national studies from the UK and Australia and limits the generalizability of findings to times of public health crises [34,35]. The sample generalizability is potentially limited to urban centres where a greater number of calls, but also a greater number of resources are present. As mentioned in the discussion this cohort may represent a uniquely resilient cohort and those who had already quit/gone on a leave of absence or not responded may have done so due to high levels of burnout. A limitation is the fact that we relied on managers to distribute the study to participants, which did not occur at times for various reasons, as listed in the results. Considering the relatively small sample size, it is possible that we did not reach saturation for the qualitative component and therefore a sufficient sample size. This is a key limitation of the study.

### 4.2. Future Areas of Research

Including an observational component could strengthen the findings of future studies. Also, a longitudinal approach could capture the evolving nature of burnout and mental health over time, particularly as the effects of COVID-19 subside. If implemented, the effects of any of the recommendations could also be studied in this manner. Considering actual/perceived training gaps were a major source of distress, further robust training on suicide risk assessment, difficult topics (violent/sexual abuse, aggression, manipulation), available community resources, and how to recognize and manage burnout may be of benefit as these were topics often cited in both the open-ended questions and quantitative components as leading to burnout. Further studies should address how vicarious post-traumatic growth, experience, training, work arrangements, and personal and professional lives contribute and/or moderate the observed relationships between age/work experience and CBI and ProQOL subscale scores. Future studies may benefit from a larger sample size with more diversity in order to strengthen conclusions.

## 5. Conclusions

This is the first study to study mental wellbeing and capture the proportions of various burnout and professional quality of life scores amongst crisis hotline responders across Canada and during the COVID-19 pandemic, which were relatively high. Younger responders seem to be more affected than older counterparts, which requires further research. The support and training methods offered by crisis hotline centres were acknowledged, appreciated, and used by crisis hotline responders; however, their availability was not always known, and further support was still desired. More flexibility regarding work arrangements was also highlighted by responders as a desire. Considering the increasing proportions of burnout/stress and staff turnover during this public health emergency, we feel that implementation of more tailored and robust training regarding burnout/stress and associated supportive measures is warranted. Recommendations are provided in the Supplementary Materials Section, with the aim of helping to retain responders for longer and prevent future staffing shortages, whether during times of public health emergencies or not.

## Figures and Tables

**Table 1:** Demographic characteristics.

**Average Age**	39.68 (8 participants did not answer)
**Gender**	
Female	107 (78.7%)
Male	25 (18.4%)
Neutral	1 (0.74%)
Non-Binary	3 (2.21%)
**Ethnicity** ^a^	
Arab	7 (5.0%)
Black	1 (0.7%)
Caucasian	107 (75.9%)
East Asian	8 (5.7%)
South Asian	8 (5.7%)
Southeast Asian	1 (0.7%)
Latin	4 (2.8%)
Other	5 (3.6%)
**Province**	(1 participant did not answer)
Alberta	28 (20.7%)
BC	18 (13.3%)
Manitoba	8 (5.9%)
NB and PEI	2 (1.5%)
Ontario	54 (40%)
Québec	21 (15.6%)
Saskatchewan	4 (3.0%)
**Language**	(1 participant did not answer)
English	117 (86.7%)
French	15 (11.1%)
English and French	3 (2.2%)

^a^ Totals do not equal 136 as 5 participants identified as 2 different ethnicities.

**Table 2:** Copenhagen Burnout Inventory (CBI) and professional quality of life (ProQOL) mean subscale scores with standard deviations and Cronbach’s α for the current study (Cronbach’s α was calculated to assess internal consistency reliability).

	**Current Study**
**CBI**	**Mean (SD)**
Personal Burnout	43.78 (19.61), n = 136, Cronbach’s α = 0.90
Work-Related Burnout	41.57 (18.04), n = 136, Cronbach’s α = 0.80
Client-Related Burnout	36.40 (16.13), n = 124, Cronbach’s α = 0.81
	**Current Study**
**ProQOL**	**Mean (SD)**
Compassion Satisfaction Scale	55.14 (7.97), n = 132, Cronbach’s α = 0.91
Burnout Scale	49.59 (7.80), n = 131, Cronbach’s α = 0.81
Secondary Traumatic Stress Scale	59.92 (7.00), n = 131, Cronbach’s α = 0.75

**Table 3:** Linear regressions for variables to predict CBI and ProQOL subscale scores.

	Coefficient B	SE	95% CI	*p*-Value	t-Value
**Age**					
CBI Personal Burnout	−0.43	0.09	−0.61–−0.26	0.001	−4.796
CBI Work-Related Burnout	−0.44	0.08	−0.61–−0.28	0.001	−5.249
CBI Client-Related Burnout	−0.36	0.08	−0.53–−0.20	0.001	−4.458
ProQOL Compassion Satisfaction Scale	0.13	0.04	0.05–0.21	0.002	3.237
ProQOL Burnout Scale	−0.15	0.04	−0.24–−0.06	0.001	−3.301
ProQOL Secondary Traumatic Stress Scale	−0.09	0.05	−0.18–−0.002	0.044	−2.031
**Work Experience (Months)**					
CBI Personal Burnout	−0.11	0.06	−0.22–−0.01	0.052	−1.962
CBI Work-Related Burnout	−0.08	0.05	−0.18–0.02	0.128	−1.531
CBI Client-Related Burnout	−0.11	0.05	−0.21–−0.02	0.023	−2.230
ProQOL Compassion Satisfaction Scale	0.06	0.02	0.01–0.10	0.017	2.409
ProQOL Burnout Scale	−0.06	0.03	−0.11–−0.005	0.033	−2.150
ProQOL Secondary Traumatic Stress Scale	−0.06	0.03	−0.11–−0.01	0.022	−2.328
**Average Hours Worked per Month**					
CBI Personal Burnout	0.12	0.10	−0.07–0.31	0.215	1.247
CBI Work-Related Burnout	−0.01	0.09	−0.19–0.16	0.886	−0.143
CBI Client-Related Burnout	0.06	0.08	−0.11–0.22	0.514	0.655
ProQOL Compassion Satisfaction Scale	0.06	0.04	−0.01–0.14	0.099	1.663
ProQOL Burnout Scale	−0.02	0.04	−0.11–0.06	0.603	−0.521
ProQOL Secondary Traumatic Stress Scale	0.01	0.04	−0.07–0.10	0.806	0.246
**Average Length of Day Shift (Hours)**					
CBI Personal Burnout	1.30	0.75	−0.18–2.78	0.086	1.731
CBI Work-Related Burnout	0.27	0.70	−1.10–1.65	0.694	0.394
CBI Client-Related Burnout	0.80	0.62	−0.43–2.03	0.203	1.281
ProQOL Compassion Satisfaction Scale	0.43	0.31	−0.18–1.03	0.164	1.397
ProQOL Burnout Scale	−0.16	0.34	−0.83–0.52	0.644	−0.463
ProQOL Secondary Traumatic Stress Scale	−0.14	0.34	−0.81–0.53	0.682	−0.411
**Average Length of Overnight Shift (Hours)**					
CBI Personal Burnout	−0.18	0.68	−1.56–1.19	0.790	−0.268
CBI Work-Related Burnout	−0.73	0.76	−2.26–0.79	0.339	−0.967
CBI Client-Related Burnout	−0.03	0.70	−1.44–1.38	0.968	−0.040
ProQOL Compassion Satisfaction Scale	0.23	0.31	−0.40–0.87	0.456	0.752
ProQOL Burnout Scale	−0.12	0.34	−0.79–0.56	0.723	−0.356
ProQOL Secondary Traumatic Stress Scale	−0.17	0.32	−0.81–0.46	0.59	−1.172
**Average Number of Overnight Shifts/Month**	−0.43	0.36	−1.16–0.30	0.247	0.007
CBI Personal Burnout	2.75	1.20	0.39–5.12	0.023	2.300
CBI Work-Related Burnout	1.24	1.12	−0.97–3.45	0.268	1.113
CBI Client-Related Burnout	2.36	1.04	0.29–4.42	0.026	2.261
ProQOL Compassion Satisfaction Scale	−0.01	0.50	−1.00–0.99	0.990	−0.013
ProQOL Burnout Scale	1.02	0.54	−0.05–2.10	0.062	1.886
ProQOL Secondary Traumatic Stress Scale	1.49	0.54	0.43–2.55	0.006	2.783

## Data Availability

Individualized data are unavailable due to privacy and ethical standards held by KCL.

## Supplementary Materials

The following supporting information can be downloaded at: https://www.mdpi.com/article/10.3390/healthcare13091025/s1, Table S1. Participants’ knowledge and views on available support and training methods to mitigate work-related burnout/stress (Custom made questions). Table S2. Commonly identified themes of open-ended questions. Table S3. Commonly identified themes of open-ended question #4 with breakdown by age group.
