# Supplementary material for: Burnout and Professional Quality of Life Amongst Crisis Hotline Responders: A Cross-Sectional Survey in Canada During COVID-19
Source: Healthcare (Basel). 2025 Apr 29;13(9):1025. doi: 10.3390/healthcare13091025 (PMC12071478; doi:10.3390/healthcare13091025)
Supplement: Supplementary file 1 [file healthcare-13-01025-s001.zip › healthcare-3384383-supplementary.pdf]

Table S1.

Participants' knowledge and views on available support and training methods to mitigate work-related burnout/stress (Custom made questions)

**Questions on supports available and current responder training methods to mitigate burnout/stress**				**Support methods viewed as being most helpful to mitigate burnout/stress** [a]	
	**Yes**	**No**	**I Don't Know**		**Tallied results**
Does the rigidity of your crisis hotline schedule regularly cause you stress? [b]	88 (65.7%)	39 (29.1%)	7 (5.2%)	Having a flexible schedule	83
Did your training involve a component on recognizing and managing burnout?	79 (58.1%)	41 (30.1%)	16 (11.8%)	Training on recognizing & managing burnout	62
Do you have access to ongoing training on how to manage calls?	103 (75.7%)	23 (16.9%)	10 (7.4%)	Access to ongoing training	61
Does your centre have a buddy system pairing up experienced and new responders for ongoing support?	64 (47.1%)	62 (45.6%)	10 (7.4%)	A buddy system pairing up experienced and new responders for ongoing support	59
Are you able to debrief with supervisors after a difficult call?	128 (94.1%)	4 (2.9%)	4 (2.9%)	Debriefing with supervisors after calls	54
Are responders at your centre asked to provide feedback on support desires?	75 (55.1%)	34 (25.0%)	27 (19.9%)	Having an avenue to provide feedback on support desires	51
Did you training involve a component on setting limits/boundaries with callers?	130 (95.6%)	5 (3.7%)	1 (0.7%)	Training on setting limits/boundaries with callers	50
Do you feel comfortable sharing any experiences of burnout with supervisors? [c]	96 (71.1%)	30 (22.2%)	9 (6.7%)	A comfortable environment to share experiences of burnout with supervisors	49
Are you able to debrief with peer responders after a difficult call?	99 (72.8%)	32 (23.5%)	5 (3.7%)	Debriefing with colleagues after calls	48
Do you have periodical check ins with supervisors to assess performance?	57 (41.9%)	74 (54.4%)	5 (3.7%)	Periodical check ins with supervisors to assess performance	47
Do you feel comfortable sharing any experiences of burnout with colleagues?	101 (74.3%)	22 (16.2%)	13 (9.6%)	A comfortable environment to share experiences of burnout with colleagues	44

Does your crisis hotline centre currently hold social activities for responders and supervisors?	61 (44.9%)	62 (45.6%)	13 (9.6%)	Organized social activity	36
Are you able to discuss any feedback with supervisors?	107 (78.7%)	15 (11.0%)	14 (10.3%)	Feedback and related discussion on your performance with a supervisor	34

a. An additional custom-made question asked participants to list their top 5 choices in no particular order of support methods viewed as being most helpful to mitigate burnout/stress. Each method they listed contributed to the tallied results.

Table S2.

Commonly identified themes of open-ended questions

Open-ended Question	Common Themes Identified	Number of times theme identified
Which of the support methods available to you do you feel help the most and why? (n=121)	Support from staff	74
	Peer support	31
	Further training	13
	Self-care	7
What do you think contributes the most to your stress as a crisis hotline responder? (n=123)	Difficult callers	46
	Feelings of inadequacy	38
	Unpredictability	16
	Rigid schedules	16
	Technology issues	12
How do you feel you could be better supported as a crisis hotline responder? If you feel that your support level is good, please state why. (n=124)	Level of support is good	54
	Support from staff	23
	Further training	21
	Improved relationships with staff	15
	In-person support	10
	Flexible schedules	8
How has burnout affected your ability to be a crisis hotline responder? (n=121)	No burnout	46
	Unable to provide best care	44
	Poor personal health	9
Do you have any other comments you would like to add which have not been discussed? (n=61)	Frustrated with the system	9
	Desire for further training	7
	Work is fulfilling	6
	Desire for improved relationships with staff	4

Table S3.

Commonly identified themes of open-ended question #4 with breakdown by age group

Age Group	No Burnout	Poor Personal Health	Unable to Provide Best Care
18-24	9	4	15
25-54	17	3	25
55-64	10	1	1
65+	10	1	3
